# Evolution of the Division of Labor between Genes and Enzymes in the RNA World

**DOI:** 10.1371/journal.pcbi.1003936

**Published:** 2014-12-04

**Authors:** Gergely Boza, András Szilágyi, Ádám Kun, Mauro Santos, Eörs Szathmáry

**Affiliations:** 1 Department of Plant Systematics, Ecology and Theoretical Biology, Institute of Biology, Eötvös Loránd University, Budapest, Hungary; 2 MTA-ELTE-MTMT Ecology Research Group, Budapest, Hungary; 3 Parmenides Center for the Conceptual Foundations of Science, Pullach, Germany; 4 MTA-ELTE Research Group in Theoretical Biology and Evolutionary Ecology, Budapest, Hungary; 5 Departament de Genètica i de Microbiologia, Grup de Biologia Evolutiva, Universitat Autònoma de Barcelona, Barcelona, Spain; University of Texas at Austin, United States of America

## Abstract

The RNA world is a very likely interim stage of the evolution after the first replicators and before the advent of the genetic code and translated proteins. Ribozymes are known to be able to catalyze many reaction types, including cofactor-aided metabolic transformations. In a metabolically complex RNA world, early division of labor between genes and enzymes could have evolved, where the ribozymes would have been transcribed from the genes more often than the other way round, benefiting the encapsulating cells through this dosage effect. Here we show, by computer simulations of protocells harboring unlinked RNA replicators, that the origin of replicational asymmetry producing more ribozymes from a gene template than gene strands from a ribozyme template is feasible and robust. Enzymatic activities of the two modeled ribozymes are in trade-off with their replication rates, and the relative replication rates compared to those of complementary strands are evolvable traits of the ribozymes. The degree of trade-off is shown to have the strongest effect in favor of the division of labor. Although some asymmetry between gene and enzymatic strands could have evolved even in earlier, surface-bound systems, the shown mechanism in protocells seems inevitable and under strong positive selection. This could have preadapted the genetic system for transcription after the subsequent origin of chromosomes and DNA.

## Author Summary

The RNA world refers to the stage of early evolution when RNA macromolecules were responsible both for storing hereditary information and performing enzymatic activities. Conflict arises between these two functions, however, as enzymatic activities of the ribozymes are in tradeoff with their replication rates. Here we address this problem by investigating the evolutionary emergence of a primordial transcription-like system in model protocells inhabited by unlinked replicators. Our numerical analysis demonstrates that division of labor between genes and enzymes could have emerged, given that there was a moderate to strong tradeoff between the enzymatic and template efficiency of one strand of the ribozymes. This division of labor results in a strong asymmetry in the numbers of the enzymatic and genetic strands of the macromolecules, in favor of the former. We offer insight into the emergence of the first transcription-like system, which is today characteristic of all known life forms.

## Introduction

The RNA world is “almost a logical necessity”, for example by the fact that aminoacyl-tRNA synthetases are not among the most ancient proteins [1]. Despite eminent attempts [2], [3] we still lack a generalized RNA replicase that would be able to unzip and copy general, long RNA templates, similar to the contemporary activity of, say, the Q*β* replicase [4], made of protein. A way out could be the assembly, out of replicable shorter pieces, of a replicase and an associated ligase [5], encouraged by the recent finding of a collectively autocatalytic ligase-based RNA network [6]. Twenty years ago the possibility of an early evolution of a division of labor between gene (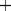) and enzymatic (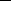) RNA strands was raised: “The fate of both the plus (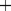) and minus (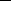) strands is important for the following discussion. If both strands are to be replicated, both of them must be recognized by the replicase: the 3′ and 5′ ends of the same strand must therefore be complementary (it is assumed that replication goes in the 5′→3′ direction as today). Interestingly, violation of such a complete symmetry opens up the possibility for a very early origin of “transcription” in the form of replication bias. If the plus strand is the gene, and the minus strand is the ribozyme, naturally it pays to make more enzymes than genes. If the tag of the minus ribozyme acts as a weaker target (owing to some point mutations, for example) for the replicase, this shift in “emphasis” is guaranteed” ([7], p. 448). The authors noted that there is such asymmetry in contemporary RNA viruses [8].

Besides their target affinity, the complementary strands of RNA molecules also have to be different regarding enzymatic activities. It is not inconceivable that complementary strands of RNAs can act as enzymes: Sergei Rodin has convincingly argued that this could have been the case for at least some tRNA [9] and aminoacyl-tRNA synthetase [10] species. It is thus biologically plausible to assume a system where both RNA strands would be weakly enzymatic, but in general this would imply different functions (unless the two strands are palindromic or the contexts in which the strands must act are highly comparable, as in the Rodin case). To conclude, a truly symmetric initial condition in enzymatic activities cannot be very common. Having said that, it is probable that one strand would lose the weak enzymatic function, whereas the complementary strand would be optimized for its enzymatic activity.

As it is likely that some surface-bound metabolic complexity preceded the advent of protocells (e.g. Ref. [11]), earliest ribozymes may also have acted on surfaces [12], [13], including evolving replicases [14]. It is in the context of such a surface-bound replicase population that the evolution of strand asymmetry has been dynamically investigated by the technique of cellular automata [15]: the authors have shown that strand asymmetry evolves (assuming a strand-displacement replication mechanism), but depending on diffusion and decay rates in a complex manner; sometimes genes rather than enzymes dominated the population. No model in the context of metabolically active ribozymes [13], [16] is known.

## Results/Discussion

**Figure 1:**
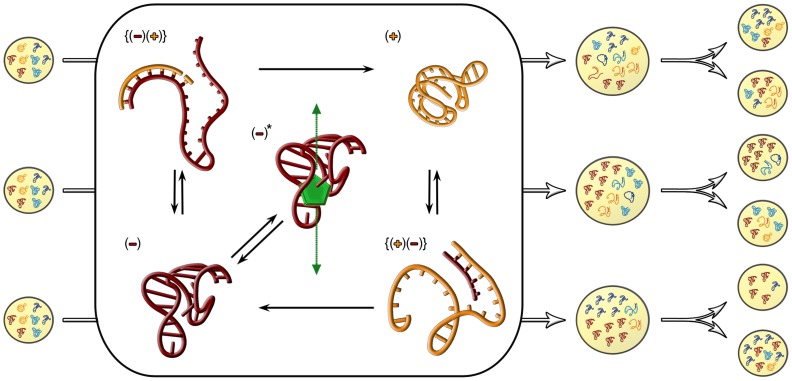
Schematic representation of the main reactions and components of vesicles with complementary replicating strands. Vesicles are composed of two types of macromolecules (type 1 as red, and type 2 as blue), and with two strand types (plus (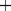) strands with light, and minus (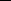) strands with dark shading). The minus (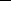) strands (molecules colored dark red) serve both as enzymes (enzymatic activity indicated with asterisk) for producing monomers (molecule colored green) from source material, and as templates for producing plus (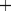) strands (molecules colored orange). The monomers are used as the building blocks (green arrow) for the productions of replicators (replication complexes are indicated in curly brackets). The plus strand only serves as template for producing minus strands. For molecule type 2, the metabolic and replication processes are similar to those of molecule type 1 described above, except that the minus (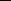) strand catalyzes a different chemical reaction.

Here we address the problem of RNA strand asymmetry in the context of metabolically active ribozymes encapsulated in reproducing protocells, relying on the stochastic corrector model [12], [17] for the basic dynamics. There are two different ribozymes (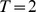) that are assumed to be essential for protocell growth and reproduction (Figure 1). In contrast to previous treatments plus (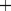) and minus (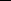) strands are explicitly considered. For simplicity we assume that only minus strands are enzymatically active. All templates grow stochastically within each protocell, and protocells also grow and divide stochastically. There is selection at two levels: faster replicating templates within protocells have an advantage, but protocells with a balanced and adequately abundant ribozyme composition are favored [17]. Although we assume their existence, we do not explicitly model replicase molecules, except that a limited number of templates can be replicated at the same time. Their effect is assumed to allow for copying of plus strand from minus strands and *vice versa*, including neat strand separation (which is still an unsolved problem in the origin of life studies [18]). It is assumed that minus strands being copied cannot perform enzymatic function at the same time, due to the opening of the catalytic sites. The two ribozymes are assumed to contribute to the production of the nucleotide monomers of the RNAs. One of the ribozymes (type 1) transforms a source material 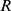 available in the environment to intermediate 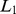, which in turn is transformed by the other ribozyme (type 2) to the monomer 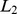. The monomer 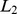 is then consumed to build up the four different kinds of strands present in the vesicle. Concrete examples of similar ribozymes that could have helped sustain the RNA world have been successfully selected *in vitro* [19], including nucleoside synthesis, phosphorylation of nucleosides, activation of nucleotides, and processive RNA primer extension. The rates of these reactions are determined by the catalytic activities of the ribozymes. The enzymatic activities of the ribozymes are in trade-off with their replication rates (e.g., active ribozymes are more difficult to unfold due to a denser structure and substrate binding), and the *relative* replication rates compared to those of complementary strands are evolvable traits of the ribozymes. Both higher and lower relative replication rates of the minus strands are allowed to evolve. The traits can change at each replication due to mutations. When the within-vesicle concentration of RNAs reaches a critical level the vesicle splits into two and its content is divided randomly, without replacement, between the two resultant daughter vesicles. See Methods for details and Table 1 for parameters and their values used throughout this study.

**Table 1:** Parameters of the model.

Parameter	Definition and value(s)
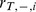	replication rate and affinity of the 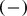 strand, 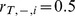
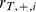	the initial replication rate and affinity of the 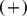 strand, 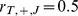
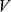	number of vesicles in the population, 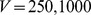
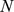	initial number of molecules per vesicle, 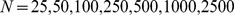
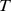	number of replicator types, 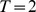
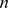	number of mutant classes, 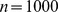
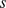	number of monomers per macromolecule, 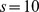
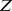	maximal number of replication complexes, 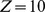
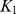	kinetic parameter of conversions 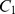 , 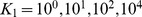
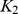	kinetic parameter of conversions 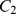 , 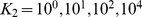
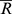	fixed concentration of the input material 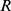 , 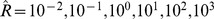
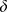	degradation rate of macromolecules, 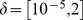
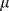	mutation rate, 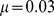 and 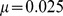
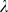	mutational variability, parameter of the Poisson distribution, 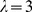
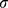	mutational variability, parameter of the normal distribution, 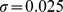
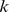	strength of trade-off, 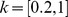

Average copy number of plus strands can be reduced through evolution even to 1 or 2 gene strands per protocell in cases when trade-off is strong between replication and enzymatic rates. The survival of plus strands in such cases is ensured by the fact that they can be copied from the ribozymes. Figure 2 shows an example of such a successful division of labor between enzymes and genes. In Figure 3 we demonstrate that evolutionary trajectories converge to the same equilibrium ratio of division of labor from different initial states, even when the evolution of replication rates and enzymatic rates is not bound, but only limited by the trade-off function assumed, and when the replication affinity of the plus strand is also allowed to evolve (Figure 3). Less pronounced division of labor is observed for weaker trade-off between replication rate and metabolic efficiency (Figure 4A), for higher numbers of molecules per protocell (Figure 4B), and for higher food concentration and kinetic rate constants (Figure 4C). By far the strongest effect is that of the trade-off, which is understandable, since it is a trait that affects every ribozyme individually. The mild decrease with protocell size is due to the fact that if there are many RNA molecules in total, there are likely to be many enzymes present anyhow, thus the force of selection should decline with protocell size. Similarly, higher food concentrations and higher kinetic rate constants reduce the force of selection for very high enzymatic efficiency. We note that some division of labor evolves even with negligible trade-off: this we attribute to the metabolic cost of the templates. In short, for the same total template copy number, protocells harboring more enzymes than genes are better off than those with reversed proportions, since the former carry a smaller load of “useless” templates (redundant genes). This effect becomes more pronounced with low food concentrations and kinetic rate constants, as in these cases the selective advantage of protocells with more enzymes increases (Figure 4C). Of course, assortment load (i.e., the drop in average fitness due to the random loss of any essential gene after stochastic assortment of templates in the two daughter protocells), and the fact that high enzymatic efficiency can already be reached without evolving high rate of strand asymmetry, prevents the system from evolving stronger asymmetry without strong trade-off.

**Figure 2:**
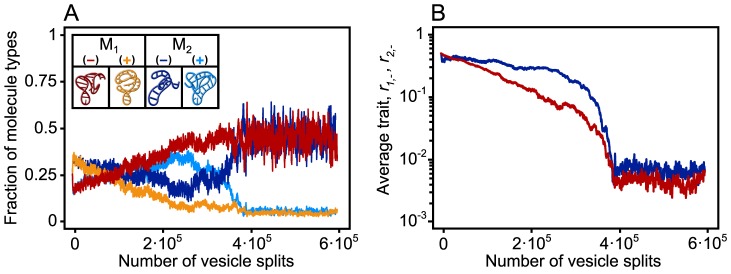
The evolution of division of labor between minus (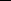) and plus (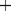) strands. (**A**) A representative example of simulations resulting in asymmetric strand separation averaged over the population of 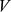 vesicles (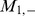: red; 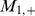: orange; 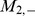: dark blue; 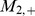: light blue). Starting from an initially symmetric state, i.e. all strand types are represented in equal numbers (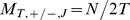), and of equal replication rates (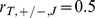) (*J* denotes the mutation class with trait 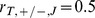). The trade-off in this case is assumed to be strong between the replication affinity and the catalytic activity. Hence the trait 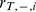 of the minus strand (**B**) gradually evolves towards lower replication rates (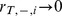) in order to achieve higher metabolic activity (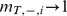). During trait evolution the ratio of minus (dark shadings) and plus (light shadings) strands changes, and the minuses significantly increase in numbers. At stable equilibrium, for the very extreme cases, only 4–8% of the macromolecules, on average 2 or 3 per vesicle, are plus strands. Other parameters: 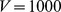, 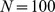, 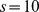, 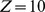, 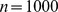, 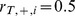, 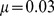, 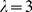, 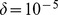, 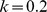, 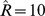, 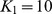 and 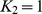.

**Figure 3:**
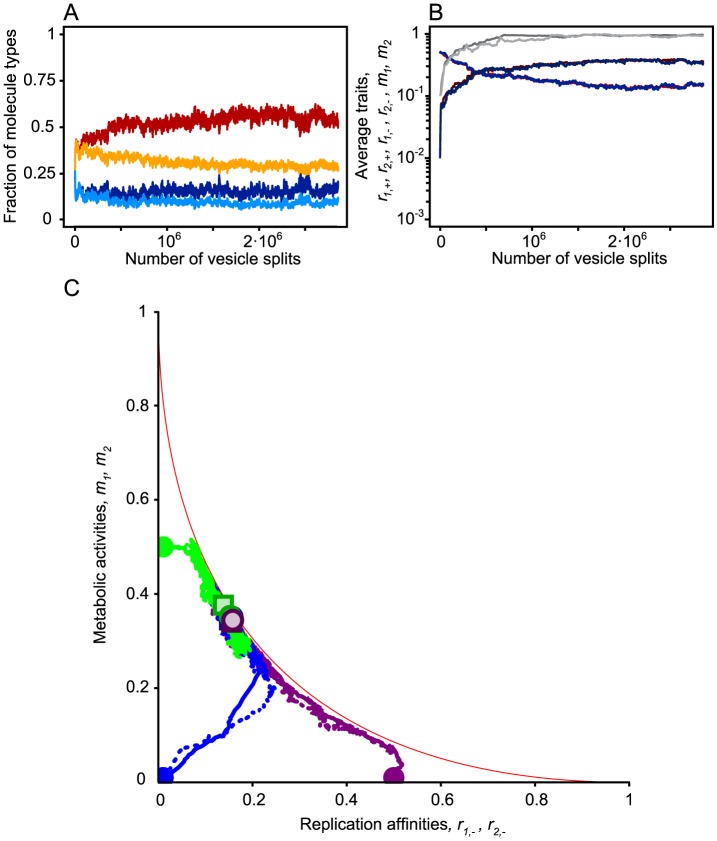
The evolution of division of labor when both replication affinity and metabolic activity of replicators are allowed to evolve separately. (**A**) A representative example of simulations resulting in asymmetric strand template reaction averaged over the population of 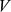 vesicles (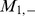: red; 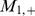: orange; 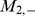: dark blue; 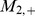: light blue). Simulations begin from an initially symmetric state, i.e. all strand types are represented in equal numbers (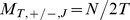) and equal template replication rates (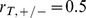). We assume low initial metabolic activity of the minus strands (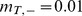) and a trade-off between the maximum values of the replication affinity and the catalytic activity of the replicators (see red line in **C**), i.e. no replicator can evolve traits above this boundary, but any rate combination below the curve is accessible (i.e. 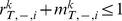, see Models Eq. 1b). (**B**) As metabolic activity gradually evolves towards high values (brown and dark blue lines, 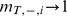) the minus strands trade in replication affinity (red and blue lines, 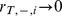) in order to reach the optimum. When the replication affinity of the plus strand can also evolve, evolution further optimizes the protocell composition in favor of strand asymmetry by evolving the highest possible affinity for the plus strand (grey and dark grey lines, 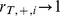). Here 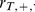 is allowed to evolve without any trade-off (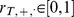, and the initial condition is 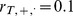). (**C**) Trajectories from different initial conditions (green: 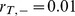 and 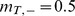; purple: 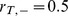 and 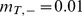; and blue: 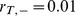 and 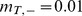) converge to the same equilibrium. Solid and dotted lines depict molecule types 1 and 2, respectively. Filled circles represent the initial data points, while light shaded circles and rectangles represent the evolutionary endpoints for traits of molecules 1 and 2, respectively. For the above results we employed a continuous-trait model, in which traits were allowed to change continuously between 0 and 1, and mutant traits were drawn from a normal distribution with the resident trait as a mean and with variance 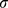. Other parameters: 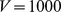, 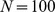, 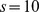, 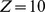, 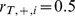, 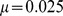, 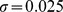, 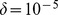, 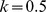, 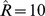, 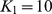 and 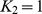.

**Figure 4:**
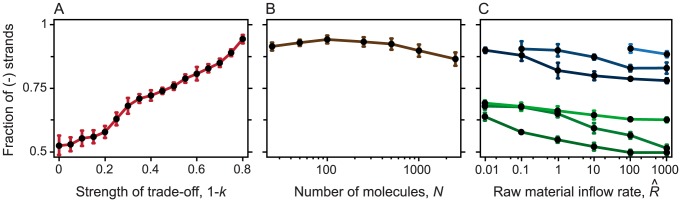
Factors affecting the rate of asymmetry between the minus and the plus strands. (**A**) In cases when the strength of trade-off is high (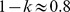), the asymmetry between the minus and plus strands is strong, however as the strength of trade-off decreases (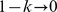), since in these cases molecules can achieve high metabolic activity without trading off their replication affinities, the asymmetry becomes less pronounced. (**B**) As the number of the initial number of molecules (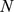) per vesicle is increased (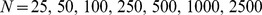) the rate of asymmetry gradually decreases (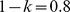). (**C**) The effect of kinetic parameters for strong trade-off (blue lines: 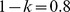) and for weak trade-off (green lines: 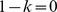). Here we increased the inflow rate of source material from the environment into the vesicle (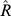) (light blue and green lines: 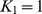 and 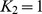; middle dark blue and green lines: 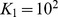 and 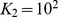; dark blue and green lines: 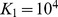 and 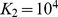). For low inflow rate and kinetic constants, high metabolic activities of minus strands evolve, which results in high rate of asymmetries between the two strands. However lowering the inflow rate or the kinetic rate of reactions beyond a threshold results in the extinction of replicators (notice the absence of equilibrium ratio of asymmetry, for example 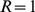, 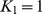 and 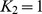, i.e. left hand side of the light blue curve). The results are averaged over 5 replicate model runs, and over 1,000,000 molecular update steps after reaching equilibrium. Whiskered bars represent the standard errors of the replicate runs. Other parameters (if not stated otherwise): 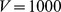, 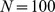, 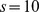, 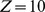, 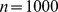, 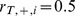, 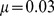, 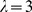, 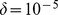, 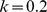, 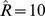, 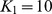 and 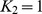.

**Figure 5:**
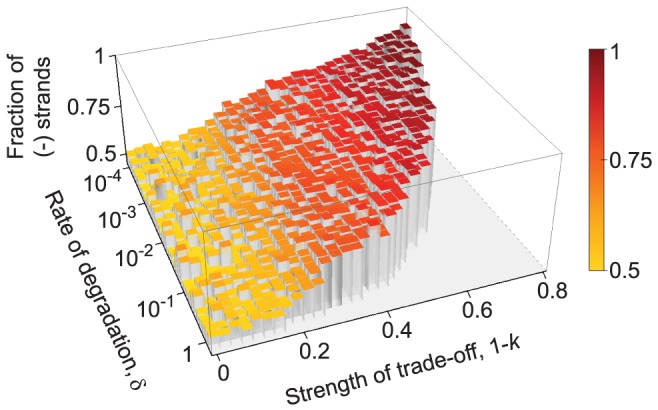
The effect of degradation rate of macromolecules on strand asymmetry. The equilibrium ratio of the minus and plus strands (indicated by the heights as well as the colors of the bars; red: 0.9→yellow: 0.5) is not affected significantly by the rate of degradation, however increasing the degradation rate above a threshold results in the extinction of the replicators (notice the flat grey area on the right hand side of the graph). For strong trade-off (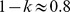), this threshold is at a lower rate of degradation, whereas higher degradation rates are tolerated as the strength of trade-off decreases (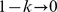). The results are averaged over 3 replicate model runs. Other parameters: 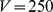, 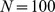, 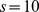, 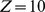, 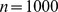, 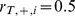, 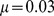, 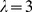, 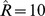, 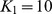 and 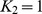.

High degradation rates can narrow the potential for the evolution of pronounced division of labor. Extreme trade-off between replication and metabolic activity selects for only few gene strands per protocell, hence a higher degradation rate easily eliminates them, and the few new genes synthesized from the ribozymes as templates may well suffer a similar fate: in the end the ribozymes cannot increase in number either, so all in all higher degradation rates lead to weaker admissible trade-off and result in weaker strand asymmetry (Figure 5). Larger protocells could, however, survive at higher degradation rates, potentially allowing for strand differentiation at strong trade-offs (the lower right part of Figure 5, where populations do not survive at the parameter values employed).

Division of labor between genes and enzymes can only be partial in our model, as expected in an RNA-based system, since a complete replication cycle requires that both strands act as templates to some extent. Division of labor implies that entities required to perform two different tasks end up with one doing (mostly) one of the tasks while the other do the other task. In our case this means that one strand acts mostly as an enzyme, while the other acts mostly as an information carrier. As only one of the strands in our model has enzymatic activity, that strand can be called an enzyme. Both of the strands need to act as templates, otherwise information is lost, but as one of the strands mainly acts as template and does not have enzymatic activity, we can call this a gene.

We would like to note here, that division of labor does not require sharp, and full specialization in different tasks: intermediate degree of specialization with the interchangeability of task-performing entities suffices too [20]. The important point is that the gain in performance due to specialization in one task exceeds the cost of loss of performance due to specialization in another task [21]. Let us give two unrelated examples from biology to illustrate our point. In eusocial insects, such as bees, division of labor often implies high degree of specialization in different tasks, as for example the queen and the workers are quite different in morphology and in behavior. But among the workers there are behavioral, but no morphological, castes, groups that tend to perform different task (cleaning, foraging, tending the young, etc.) [22]. Moreover, in primitively eusocial wasps, like the *Ropalidia marginata*, even the queen cannot be distinguished from others except for the presence of well-developed ovaries [4]. The other example comes from clonal plants (such as strawberry), where the members are connected physiologically. In these plants, one “plant member” (called the ramet) may specialize in the uptake of belowground nutrients, and thus develop an extensive root system, while the other specializes in the capture of light and develops bigger leaves [5]. Only the relative investments change into shoot or root, but ramets still have both functioning root systems and leaves. In biology, it is thus common to observe intermediate levels of division of labor and functional specialization within the boundaries set by physiological and developmental constraints of an organism.

Chemical difference between the enzymes and the templates is not a requirement for division of labor between genetic and enzymatic functions. The present neat chemical distinction between genes (DNA) and enzymes (proteins) is a rather late invention. Comparative analysis of the genes involved in DNA replication [1], [2] and the age of protein domain fold required for dNTP synthesis [3] suggest that the emergence of DNA genome was a late phenomenon which could have happened after the LUCA, thus was most likely a successor to the RNA world. As the authors of a somewhat related theoretical work note: “DNA releases RNA from the trade-off between template and catalyst that is inevitable in the RNA world and thereby enhances the system's resistance against parasitic templates” [23] (p. 2). It is exactly this trade-off that drives the evolution of the division of labor in our protocellular system. (We note in passing that the analysis in Ref. [23] is not enough by itself to explain the advantage of DNA, since DNA molecules can also be selected to act as enzymes [24]).

**Figure 6:**
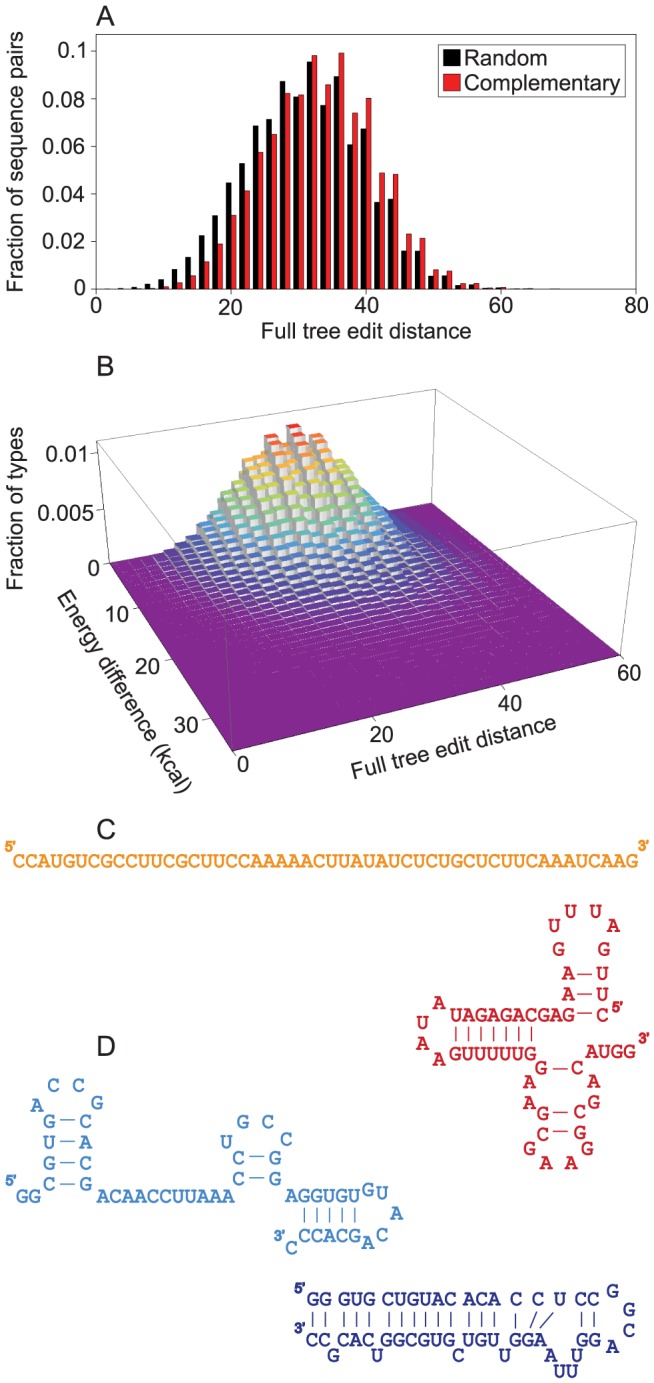
Characteristics of secondary structures of complementary strands. The characteristics of minimum free energy secondary structures are measured on a sample of 10^7^ randomly generated sequences of length 50. In case of complementary strands, the complementary sequences of the randomly generated strands are also analyzed. (**A**) Complementary strands have higher full tree edit distance between them (red bars) than random sequence pairs (black bars). (**B**) Energy difference between members of pairs of complementary, folded strands. Around tree edit distance 30 most complementary, folded structures have negligible energy difference, but a decreasing proportion of pairs show a difference of up to 40 kcal. (**C**) Example of a complementary pair of strands in which one of the strands does not have a structure, while the other has a rich structure. The difference of their minimum free energies is (6.6 kcal). (**D**) Example of a complementary pair of strands in which the two strands have very different (tree edit distance 68) but still rich structures. The difference of their minimum free energies is (7.0 kcal).

We investigated the evolution of division labor between enzymatic and genetic strands based on the implicit assumption that minus and plus strands can have very different secondary structures. This indeed proves to be the case: on a sample of 10 million sequences, the distances between the secondary structures of minus and plus strands are slightly higher than those between pairs of randomly generated sequences (Figure 6A). Furthermore, there is asymmetry in the complexity of secondary structures (Figure 6A, C, D); and the difference between the free energies of folding can reach levels up to 20 kcal/mol (Figure 6B). Thus, there is a fraction of complementary, folded strand pairs for which one member is more readily opened by a replicase than the other, due to the looser structure of the former (Figure 6C). Here we have only considered the minimum free energy (MFE) structures of the RNAs. It is known that there are suboptimal structures that could be quite close energetically to the MFE structure [25], and thus provide additional ways in which the two strands can be different (albeit evolution can lead to well-defined structures with little ambiguity in their energetically close sub-optimal structures [26]). Co-folding of the RNA with smaller RNAs can further increase the structural diversity of RNAs [27], again possibly promoting functional diversification of the strands. Our conservative estimate of structural difference is sufficient for strand separation, and incorporation of further mechanisms can further foster the effect demonstrated above.

The origin of basic genetic operations, including replication and transcription, belongs to the key questions of the origin of life. While there has been considerable progress with template copying [2], [3], unzipping remains an open problem [18] (but see [28]). In this paper we have shown that once evolution had reached the stage of reproducing compartments with unlinked ribozymes inside, division of labor between enzymatic and gene strands readily followed provided there was moderate to strong tradeoff between the enzymatic and template efficiency of ribozymes. This is to be expected due to the tightly folded structure of ribozymes (for example the Q*β* replicase replicates the X-motif ribozyme [29] very slowly compared to other, less complex secondary structures; A. Griffiths, personal communication). Furthermore, analysis of the minimum free energy structures of real ribozymes and aptamers indicates that there is a tendency of them being more thermodynamically stable than random sequences (81.9% are more stable than half of the random sequences; 59.6% are more stable than 75% of the random sequences; and 27,5% are more stable than 95% of the random sequences). This transcription-like process could have been augmented by the evolution of tags recognized by the replicase as envisaged by Szathmáry and Maynard Smith [7], although we have not included this component in the present model. We conclude that division of labor between genes and enzymes was under strong positive selection in the RNA world.

## Methods

### Characteristics of the RNA molecules

An RNA molecule 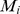 of length 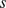 (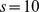) is characterized by its type 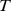 (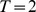), its role of being a ribozyme (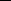) or an informational strand (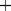), and a combined trait 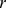 representing the replication affinity and the polymerization rate of the given molecule. We assume 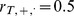 for the (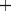) strands (except for Figure 3, in which case 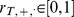). The traits 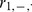 and 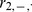 (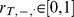) are the evolvable traits of our model.

The ribozymes catalyze reactions with metabolic activity 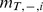. We assume that the ribozymes cannot perform any metabolic function during the replication process, as the molecule is in an unfolded state and cannot form the pocket responsible for enzymatic activity. Thus there is a trade-off between the processes of replication and catalytic activity which is characterized by the following one-parameter function
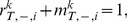
(1a)and for additional investigations (see Fig. 3) we also allow
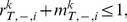
(1b)where 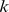 characterizes the strength of this trade-off (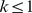).

Mutation can occur with probability 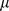 at each replication of the molecules. We allow 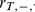 to change in a discrete manner:
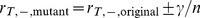
(2)where 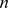 is the number of mutation classes and 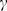 is randomly drawn from Poisson-distribution with parameter 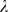. There is an equal probability of having mutants with higher or with lower traits compared to the original trait. We opted for discrete traits as it facilitates faster convergence to evolutionary equilibrium, as our additional studies indicated similar result can be attained employing continuous traits (see Fig. 3). In the latter case traits are allowed to change on a continuous scale, and mutant traits are drawn from a normal distribution with the resident trait as a mean and with variance 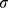.

### Chemical reactions in the vesicles

Reactions involving the macromolecules 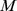 fall into four classes: (1) catalyzed conversion 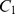 of the raw material (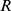) into the intermediate (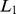); (2) catalyzed conversion 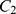 of the intermediate (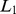) into the monomer (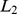); (3) polymerization 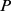 of a new strand; and (4) degradation 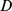 of a macromolecule. As there are 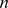 mutational classes, the trait 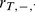 (and thus 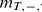) can have 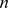 different values. Accordingly, the total number of possible reactions are: 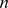 conversions 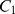, 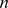 conversions 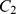, 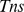 polymerization 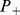 reactions involving the plus strands as templates, 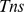 polymerization 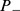 reactions involving the minus strands as templates, and 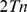 reaction of degradation 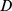.
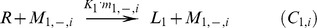


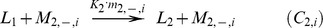


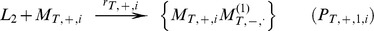


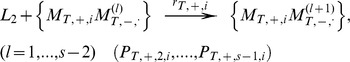


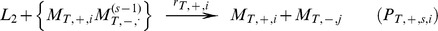


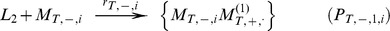


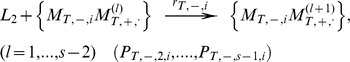


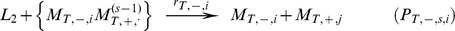


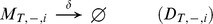


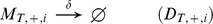
where 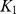, 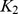 and 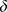 are kinetic constants for the corresponding reactions, 
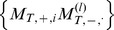
 or 
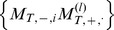
 denotes the complex involving a template strand and the an intermediate forms of the complementary strand consisting of 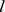 monomers (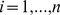, 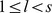).

The full replication cycle is completed after two steps of copying: 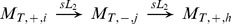. We assume a limited number of replicase enzymes in a vesicle, hence we limit the number of simultaneous replication processes to 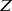.

We apply the Gillespie algorithm [30] to follow the reactions within the vesicle. We introduce the quantity 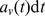 that characterizes the probability of reaction 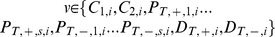 (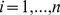, 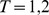) in the time interval 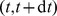. 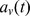 is the product of two factors: the chemical constant for the given reaction type 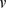 and the number of possible reactions within a given vesicle. For the reaction 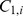


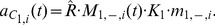
(3)We note that the input material 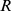 has a fixed concentration 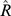. Similarly, for reaction types e.g. 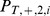 and 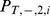


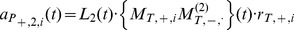
(4)

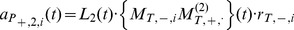
(5)


Degradation is a monomolecular reaction; its probability is proportional to the present amount of the given molecules. The chemical constants 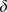 for degradation is common for all types of macromolecules 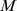. The degradation and dissociation of replication complexes is neglected in our model.

We define the sum of all 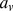
's as
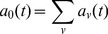
(6)


The time 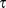 after 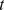 at which the next reaction will take place is drawn from an exponential probability density function of rate 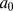:
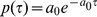
(7)


At time 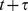, we choose reaction 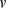 as the next reaction with probability 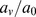 in the vesicle. We then update the number of different molecules according to reaction scheme 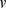 and the process is reiterated.

### Population dynamics of protocells

The population is composed of 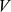 number of protocells, with the initial number of 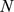 replicating molecules, and 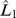 number of intermediate and 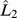 number of building block molecules. The number of RNAs can increase up to 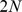, at which point the vesicle splits randomly assorting all the replicator molecules into two daughter vesicles. During splitting, small molecules 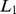 and 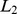, as well as the initiated replication complexes are also randomly allocated to the daughter vesicles. One daughter vesicle is replacing the parent, while the other replaces another random vesicle in the population (i.e. it is a Moran process [31]).

### Structural similarity of complementary strands

We have assumed that complementary strands can be quite dissimilar in structure, so that one of them can fold to be a ribozyme while the other has a structure that can be more readily processed by the replicase enzyme. We check if complementary strands can be dissimilar enough to potentially achieve such a state. We have determined the minimum free energy structure of 10^7^ random RNA sequences of length 50. We have also determined the minimum free energy (MFE) structure of 10^7^ random complementary pairs of RNAs of length 50. Each individual sequence's structure is compared to the structure of the next sequence to obtain the full tree edit distance [32] between the two structures. Similarly, the distance between each complementary pair of sequences is also determined. All computations are done with the Vienna RNA Package 2.0.7 [33].

### Thermodynamic stability of ribozymes and aptamers

We analyzed the set of 305 ribozyme and aptamer sequences mainly from the Aptamer Database [34] and from the review of Chen and co-workers [35] (the full list is reported in Supplementary Table S1 of [36]). For each sequence the MFE was determined. Then we generated 100,000 random sequences of the same length and recorded their MFE. Then we counted the number of random sequences having lower MFE (i.e. being more stable) than the ribozyme/aptamer.
